# The Risk of Venous Thromboembolism and Ischemic Stroke Stratified by VTE Risk Following Multiple Myeloma: A Korean Population-Based Cohort Study

**DOI:** 10.3390/jcm13102829

**Published:** 2024-05-11

**Authors:** Hyun Jin Han, Miryoung Kim, Jiyeon Lee, Hae Sun Suh

**Affiliations:** 1 Department of Regulatory Science, Graduate School, Kyung Hee University, Seoul 02447, Republic of Korea; hyunjin.han@khu.ac.kr (H.J.H.); jiyeon.lee@khu.ac.kr (J.L.); 2 Institute of Regulatory Innovation through Science, Kyung Hee University, Seoul 02447, Republic of Korea; miryoung.kim@khu.ac.kr; 3 College of Pharmacy, Pusan National University, Busan 46241, Republic of Korea; 4 College of Pharmacy, Kyung Hee University, Seoul 02447, Republic of Korea

**Keywords:** venous thromboembolism, ischemic stroke, multiple myeloma, IMPEDE VTE

## Abstract

**Background**: Multiple myeloma (MM) is associated with high morbidity and mortality, with elevated rates of arterial thrombosis and venous thromboembolism (VTE) and ischemic stroke (IS). We aimed to estimate the incidence of VTE and IS categorized by the VTE risk grade among individuals with MM in Korea. Additionally, we explored the potential of the IMPEDE VTE score as a tool for assessing IS risk in patients with MM. **Methods**: This retrospective cohort study comprised 37,168 individuals aged ≥ 18 years newly diagnosed with MM between January 2008 and December 2021 using the representative claims database of the Korean population. The risk of the incidence of VTE and IS within 6 months after MM diagnosis was stratified based on high-risk (IMPEDE VTE score ≥ 8) and low-risk (<8) categories. The hazard ratios (HRs) were estimated using Cox proportional hazard models. **Results**: The VTE incidence was 120.4 per 1000 person-years and IS incidence was 149.3 per 1000 person-years. Statistically significant differences were observed in the cumulative incidence rates of VTE between groups with high and low VTE scores (*p* < 0.001) and between individuals aged ≤ 65 years (*p* < 0.001) and those with a Charlson comorbidity index (CCI) ≥ 3 compared to lower scores (*p* < 0.001). Additionally, the cumulative incidence rate of IS differed significantly across all groups (*p* < 0.001). The HR for the high-risk group in VTE and IS occurrence was 1.59 (95% CI, 1.26–2.00) and 3.47 (95% CI, 2.99–4.02), respectively. **Conclusions**: It is important to screen and manage high-risk groups for the early development of VTE or IS in patients with newly diagnosed MM.

## 1. Introduction

Multiple myeloma (MM) is a clonal plasma cell tumor associated with significant morbidity and mortality [1]. Arterial thrombosis and venous thromboembolism (VTE) occur at notably elevated rates in patients with MM [2,3]. Given that patients with MM having VTE exhibit a mortality rate 2.9 times (95% confidence interval [CI], 2.4–3.5) higher than those without VTE, it is imperative to identify individuals at a heightened risk for VTE and implement thromboprophylaxis in such high-risk cohorts [4].

The IMPEDE VTE score is a risk assessment model recommended by the National Comprehensive Cancer Network for predicting VTE in patients [5]. This model offers a structured approach for risk stratification by assigning weights to various VTE risk factors, thereby aiding in the initiation of chemotherapy for patients with MM. Its efficacy in predicting the VTE risk within a six-month timeframe has been validated in extensive cohort studies of American and Chinese populations [6,7].

Moreover, ischemic stroke (IS), a form of arterial thrombosis, presents a significantly higher mortality rate among patients with MM, reported at 3.4 times (95% CI, 3.0–3.8) higher compared to those without IS [4]. Despite the necessity for prior risk assessment, a substantial research gap persists in evaluating the risk of IS in patients with MM. Although general stroke assessment tools are available, they lack specificity for patients [8]. Considering the noted similarity in the risk factors between IS and VTE and the established association between IS and VTE (Odds Ratio [OR], 1.22; 95% CI, 1.12–1.33), there is a pressing need to investigate the potential utility of employing the IMPEDE VTE score to forecast the risk of IS development in patients with MM [2,9].

This study aimed to assess the incidence of VTE and IS across different VTE risk levels and evaluate the effectiveness of the IMPEDE VTE score in managing the risk of ischemic stroke. Our goal was to provide real-world evidence to support thrombosis prevention strategies in high-risk patients with MM.

## 2. Materials and Methods

### 2.1. Data Source

We conducted a retrospective cohort study using the Health Insurance Review and Assessment (HIRA) database, which covers approximately 98% of South Korea’s total population [10]. The HIRA database contains hospital claim information, including diagnosis codes (International Classification of Diseases 10th, ICD-10), dates of diagnosis, procedures, surgical history, and prescribed medications. We analyzed the HIRA database from January 2007 to December 2022. This study adhered to the Declaration of Helsinki and was approved by the Institutional Review Board of Kyung Hee University, which granted an exemption from ethical review based on the observational nature of the study as secondary database research (Approval Number: KHSIRB-22-287-2(EA)).

### 2.2. Study Population

The study cohort comprised individuals aged 18 years or older who were newly diagnosed with MM between January 2008 and December 2021. The index date was the first calendar date of chemotherapy administration. To ensure that the patients were newly diagnosed, a one-year washout period preceding the index date was implemented. Participants with a recent diagnosis of VTE or IS within six months preceding the index date were excluded from the analysis.

The patients were stratified into two risk categories using IMPEDE VTE scores: high-risk (≥8 points) and low-risk (<8 points) [11]. This scoring system serves as a VTE risk prediction tool, enabling clinicians to quantitatively evaluate the risk of VTE in MM patients based on 11 variables. These variables include the following: the use of immunomodulatory drugs (IMiDs); a body mass index (BMI) of 25 kg/m² or higher; recent pelvic, hip, or femur fracture; the administration of erythropoiesis-stimulating agents, doxorubicin, or dexamethasone; race; a history of VTE; the presence of a tunneled line or central venous catheter; and the pre-existing use of antithrombotic or antiplatelet medication. The baseline characteristics were assessed during 1 year prior to the index date.

The variables used to calculate the IMPEDE VTE scores were defined based on the established protocols outlined in previous studies [6,11,12,13,14,15]. These variables were identified using the diagnostic or Anatomical Therapeutic Chemical codes provided by the World Health Organization. The IMiDs include thalidomide, lenalidomide, and pomalidomide. Due to the unavailability of BMI information, the BMI data were defined as the diagnosis of obesity (ICD-10 code: [E66]). High-dose dexamethasone was defined as a dose exceeding 160 mg every four weeks. The detailed information regarding variable collection is provided in Supplementary Table S1.

### 2.3. Outcomes

The outcome of interest was the incidence of VTE or IS within 6 months of the index date, categorized by risk grade. VTE was defined using the following ICD-10 codes: I26, I80, I81, I82, O082, O223, O871, and O882, while IS was identified by code I63 [14,16,17]. The follow-up period for these outcomes was from one day after the index date to the earliest occurrence of the first VTE or IS event, death, 6 months after the index date, or December 2022. The outcome was measured by calculating the incidence rate per person-month, derived from the total number of new cases of an event divided by the sum of person-times. Furthermore, the hazard ratio (HR) was estimated to investigate the factors influencing the incidence of VTE or IS.

### 2.4. Statistical Analysis

Categorical variables were analyzed using either the chi-square test or Fisher’s exact test. Continuous variables were reported as the mean (standard deviation, SD) and compared using the t-test or Wilcoxon–Mann–Whitney test. The cumulative incidences were calculated using person-years per 1000 persons via the Kaplan–Meier method and compared using the log-rank test. We used Cox proportional hazards models to examine the association between high IMPEDE VTE scores and the time to event. These models were adjusted for covariates, such as sex, age, and Charlson comorbidity index (CCI) scores [6,11,12,13,14,15]. Comorbidities were recorded from one year to one day before the index date. The Concordance index (C-index) was employed to assess the predictive accuracy of survival models, such as the Cox proportional hazards model.

The statistical significance was set at *p* < 0.05, and the confidence intervals were estimated at the 95th percentile. All statistical analyses were two-sided and were conducted using the SAS Enterprise Guide version 7.1 (SAS Institute Inc., Cary, NC, USA).

## 3. Results

### 3.1. Baseline Characteristics of Population

This retrospective cohort study included 37,168 patients with NDMM after excluding ineligible patients. Of these, 1910 (5.1%) patients experienced a VTE event, and 2358 (6.3%) patients had IS during the follow-up period. The mean follow-up duration was 5.12 months (95% CI, 5.10–5.14). The average age at MM diagnosis was 65.9 years (95% CI, 65.76–66.02), with the majority (*n* = 21,638, 58.2%) aged ≥ 65 years. The baseline characteristics of the patients are summarized in Table 1.

Over six months after the initial MM diagnosis, 4268 VTE and IS events were examined. The incidences of VTE and IS were 5.1% (1910 patients) and 6.3% (2358 patients), respectively. Patients with VTE and IS events were significantly older—68.0 years (SD 13.2) and 71.5 years (SD 11.1), respectively—than those without events, whose age was 65.4 years (SD 13.2) (*p* < 0.0001). The average CCI was 4.32 (SD, 3.1) across the total population, with significant differences in the CCI scores among event-free, VTE, and patients with IS (*p* < 0.0001). The most common comorbidity in the study population was hypertension (62.5%), and the comorbidity rate was significantly different among the three groups (*p* < 0.0001).

The overall mean IMPEDE VTE score was 1.78 (95% CI, 1.76–1.81), showing significant variance among the subgroups (*p* < 0.0001). The group with IS events had the highest average IMPEDE VTE score of 2.7 (SD, 3.8), followed by the VTE event group next at 1.81 (SD, 3.3). An IMPEDE VTE score of 8 or above was present in 815 (2.5%) of the event-free patients, 76 (4.0%) of the VTE group, and 193 (8.2%) of the IS group. The use of immunomodulatory drugs was notably higher in the VTE group (25.4%, *n* = 486) compared to 15.5% (*n* = 5108) in the event-free group and 7.4% (*n* = 174) in the IS group (*p* < 0.0001). Additionally, a history of VTE prior to MM diagnosis was significantly more common in the IS group (50.4%, *n* = 1188) and the VTE group (11.9%, *n* = 228) than in the event-free group (3.1%, *n* = 1022; *p* < 0.0001).

### 3.2. Cumulative Incidence of VTE and IS

#### 3.2.1. Cumulative Incidence of VTE

**Figure 1:**
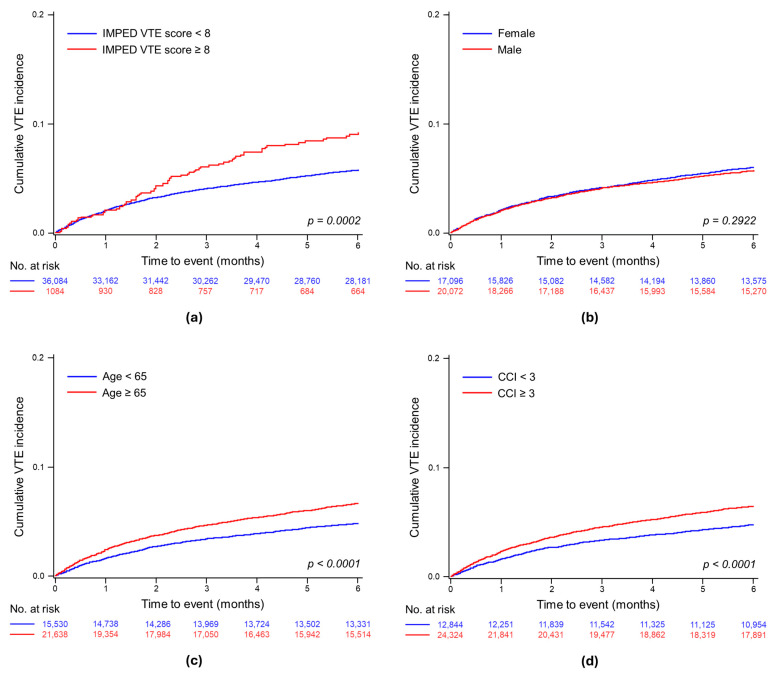
Cumulative incidence of venous thromboembolism within 6 months from diagnosis of multiple myeloma: (**a**) stratified results by VTE-IMPEDE high score level; (**b**) stratified results by sex; (**c**) stratified results by age ≥ 65 years; and (**d**) stratified results by CCI level.

The median follow-up for VTE patients was 1.51 months (95% CI, 1.95–2.10). The 6-month cumulative incidence of VTE following the initial MM diagnosis was 5.14% (95% CI, 4.92–5.37), translating to an incidence rate of 120.4 per 1000 person-years. Significant differences in the cumulative incidence rates of VTE were observed between groups with high and low IMPEDE VTE scores (*p* < 0.001), those aged ≤ 65 years (*p* < 0.0001), and those with a CCI ≥ 3 compared to lower scores (*p* < 0.0001) but not between sexes (*p* = 0.292) (Figure 1). The estimated 6-month cumulative incidence of VTE was 8.6% for the IMPEDE score ≥ 8 group versus 5.6% for the lower score group, 6.4% for patients aged ≥ 65 years versus 4.7% for younger patients, and 6.2% for the CCI ≥ 3 group versus 4.6% for those with a lower CCI.

#### 3.2.2. Cumulative Incidence of IS

**Figure 2:**
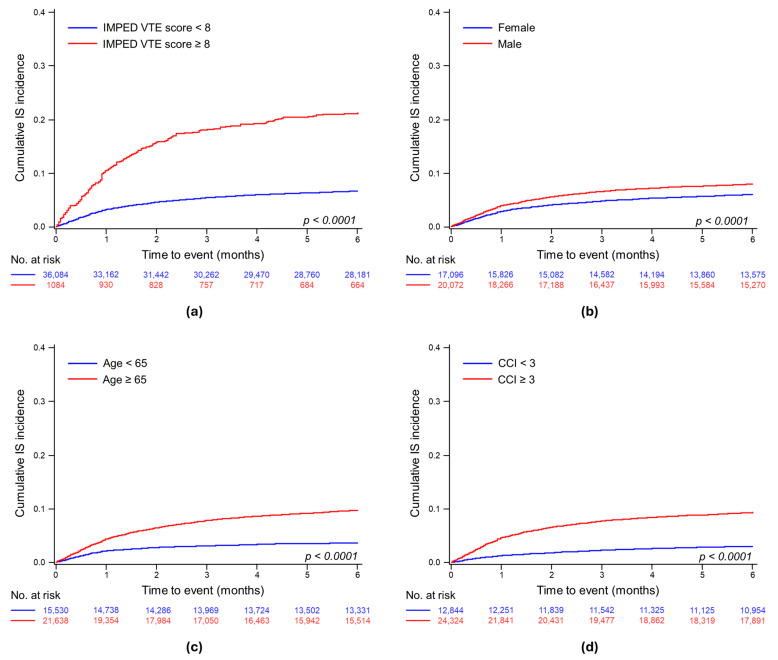
Cumulative incidence of ischemic stroke within 6 months from diagnosis of multiple myeloma: (**a**) stratified result by VTE-IMPEDE high score level; (**b**) stratified result by sex; (**c**) stratified result by age ≥ 65; and (**d**) stratified result by CCI level.

The median follow-up for patients with IS was 1.0 month (95% CI, 1.50–1.61). The 6-month cumulative incidence of IS from the first MM diagnosis was 6.34% (95% CI, 6.10–6.60), with an incidence rate of 149.3 per 1000 person-years. Significant differences in the cumulative IS incidence rates were noted between the high and low IMPEDE VTE score groups (*p* < 0.0001), sexes (*p* < 0.0001), ages ≥ 65 years and younger (*p* < 0.0001), and a CCI ≥ 3 compared to lower (*p* < 0.0001) (Figure 2). The 6-month cumulative incidence of IS was 19.0% in the IMPEDE score ≥ 8 group versus 6.4% in the low score group, 7.6% for males versus 5.8% for females, 9.1% for those aged ≥ 65 years versus 3.5% for younger individuals, and 6.2% for the CCI ≥ 3 groups versus 4.6% for those with a lower CCI.

### 3.3. Stratified Risks of VTE and IS Using IMPEDE VTE Scores

#### 3.3.1. Assessing the Impact of the IMPEDE VTE Score on the Venous Thromboembolism Risk

Our multivariate Cox hazard analysis showed significant associations between the risk of VTE events and the IMPEDE VTE score and population characteristics across the four models (Table 2). The high IMPEDE VTE score (≥8) is consistently associated with an increased risk for VTE across all models. Patients with a high IMPEDE VTE score had a 54–62% higher risk of experiencing a VTE event than those with a lower risk, with HRs ranging from 1.54 in Model 1. Even after adjusting with other population characteristics, the high IMPEDE VTE score was significantly related to the VTE event risk shown in HR 1.54 (95% CI, 1.23–1.94) in Model 2 to 1.62 (95% CI, 1.29–2.04) in Model 3 and 1.59 (95% CI, 1.26–2.00) in Model 4 (*p* < 0.0001).

Sex appeared to have no significant effect on the risk of VTE in any model. Age was another critical factor, with individuals aged ≥ 65 years facing a significantly increased risk of VTE events with HRs of 1.40 (95% CI, 1.27–1.53, *p* < 0.0001) in Model 3 and 1.34 (95% CI, 1.22–1.47, *p* < 0.0001) in Model 4. Moreover, a higher CCI score (≥3) was significantly associated with an increased risk of VTE in Model 4, with an HR of 1.29 (95% CI, 1.17–1.42, *p* < 0.0001), suggesting that patients with more significant comorbidities have a higher risk of experiencing VTE events.

The discriminative ability of the IMPEDE VTE score in predicting VTE events was further evaluated, yielding a C-index of 0.524. This result suggests the utility of the score in identifying patients at a higher risk of VTE, although the C-index indicates moderate discriminative power.

#### 3.3.2. Assessing the Impact of the IMPEDE VTE Score on the Risk of Ischemic Stroke

We explored the impact of various determinants on the incidence of IS events, and the Cox analysis results are presented in Table 3. Our investigation yielded significant insights into the associations of a high IMPEDE VTE score, sex, older age, and a high CCI score with the risk of IS.

A notable finding was the substantial link between a high IMPEDE VTE score (≥8) and an elevated IS risk across all models. The HRs, in particular, demonstrated that patients with a high IMPEDE VTE score (≥8) are at a significantly increased risk of IS events, with HRs of 3.23 (95% CI, 2.79–3.75) in Model 1, 3.22 (95% CI, 2.78–3.73) in Model 2, 3.72 (95% CI, 3.21–4.31) in Model 3, and 3.47 (95% CI, 2.99–4.02) in Model 4.

Upon regulating the population characteristics and their association with IS, we found that sex, older age, and a higher CCI significantly increased the IS risk. Sex was another significant predictor, with male patients showing a higher risk of IS events. The HRs for males were consistent across the models—1.33 (95% CI, 1.22–1.44) in Model 1, 1.35 (95% CI, 1.24–1.46) in Model 2, and 1.30 (95% CI, 1.20–1.41) in Model 3—all with *p*-values < 0.0001, indicating a 30–35% increased risk for males. Age, mainly ≥65 years, was identified as a crucial risk factor for IS. The analysis revealed HRs of 2.80 (95% CI, 2.51–3.05) in Model 3 and 2.42 (95% CI, 2.42–2.20) in Model 4, both statistically significant (*p* < 0.0001), suggesting that older individuals are significantly more susceptible to IS, with risks ranging from 2.42 to 2.80 times higher than younger patients. Moreover, a higher CCI score (≥3) was significantly associated with an increased risk of IS in Model 4, where an HR of 2.65 (95% CI, 2.37–2.97, *p* < 0.0001) was observed. These results underscore that patients with more significant comorbidity burdens, measured by a CCI score of 3 or higher, are over twice as likely to experience IS events than those with lower scores.

In addition, the discriminative capability of the IMPEDE VTE score in predicting IS events was evaluated, yielding a C-index of 0.592. This implies the possibility of using the IMPEDE score to identify patients at an increased risk of IS, although the moderate C-index suggests that there is room for further refinement in its predictive accuracy.

## 4. Discussion

The incidence of VTE among patients newly diagnosed with MM in Korea is approximately 120 per 1000 person-years. The risk for the high-risk group in VTE occurrence was significantly 1.59 times higher (95% CI, 1.26–2.00). Additionally, the incidence of IS among patients newly diagnosed with MM was approximately 149 per 1000 person-years, and there was a significant difference in the risk of occurrence depending on the risk of IS (HR = 3.47; 95% CI, 2.99–4.02).

The incidence of VTE in patients with MM is generally known to be approximately 10% but varies widely across studies due to factors, such as the study era, race, MM regimen, and the use of anticoagulants [3,18,19,20]. A cohort study using data from the U.S. Veterans Health Administration (VHA) reported the cumulative incidence of VTE at 6 months to be 6.5%, and an analysis linked with the VHA data and the SEER-Medicare database targeting MM patients aged > 65 years found an incidence of 8.6% [14,21]. In the Myeloma IX and XI trials conducted by the United Kingdom Medical Research Council, it was reported that patients with NDMM experienced a 6-month VTE cumulative incidence ranging from 2.2% to 20.7% depending on the MM regimen [22]. Research on patients with MM registered at 16 major academic medical centers in China identified an overall incidence of 6.1% for iMiD-related VTE [15]. The incidence of VTE reported in our study was 5.1%, which is slightly lower than the range reported in previous studies but aligns with the 5–8% range identified in a prior study analyzing a cohort of Korean patients with relapsed/refractory MM [23]. Our study was significant because of the population-level incidence of VTE in patients newly diagnosed with MM in Korea.

The occurrence of VTE significantly increases the mortality rate of patients with MM. The HRs for 1-year mortality in the Swedish cohort and 6-month mortality in the U.S. VHA cohort were 2.9 and 1.67, respectively, which were significantly higher in patients with MM and VTE [4,21]. In a recent systematic review, the risk of early death due to VTE in patients with MM was reported to be 2.27 times higher [24]. As the negative consequences of VTE on mortality are evident, it is also expected to result in long-term morbidity and to impair the quality of life, yet more studies addressing this issue in the MM population are needed [22]. With the increasing emphasis on the importance of managing VTE risk with an MM diagnosis, the major clinical guidelines recommend the use of valid stratification methods, such as the IMPEDE VTE score or the SAVED score, to manage VTE risk in patients with MM [5]. The IMPEDE VTE score is a VTE risk prediction model developed by Sanfilippo et al., aiming to improve VTE risk stratification beyond the previously used International Myeloma Working Group/National Comprehensive Cancer Network guidelines [11]. It was developed using data from 4446 patients with MM from the Veterans Administration Central Cancer Registry and was externally validated using SEER-Medicare data. Unlike the SAVED score, which is designed for patients beginning iMiD induction therapy, the IMPEDE VTE score can be used for any MM-directed therapy [25]. To date, it has been evaluated in real-world studies from various countries, including the U.S., China, France, and Brazil, demonstrating superior VTE risk prediction capabilities to previous risk stratification models [6,7,26,27]. However, previous research has been confined to either single- or multicenter studies involving a limited number of patients, and studies at the population level are notably insufficient.

In the study by Sanfilippo, the 6-month cumulative incidence of VTE was 3.3% for the low-risk group with an IMPEDE VTE score ≤ 3 and 15.2% for the high-risk group with a score ≥ 8 [11]. In France, the score was evaluated for 190 patients with NDMM registered in the MELISSE database, and the incidence of VTE in the low-, intermediate-, and high-risk groups was 5.9%, 9.4%, and 16.7%, respectively [26]. U.S.- and China-based chart review studies reported VTE incidences of 5.0%, 12.6%, and 24.1% and 3.8%, 8.6%, and 40.5% for the respective risk groups, underscoring the effectiveness of the IMPEDE score in VTE risk prediction [6,7]. Our study also estimated the VTE incidence to be 8.6% for the high-risk group with an IMPEDE VTE score ≥ 8 and 5.6% for those with a score < 8, showing a consistent pattern of increased risk with a higher score. Despite the clear trend in VTE incidence correlating with risk groups, as determined by the IMPEDE score, the variations in the incidence rates across studies suggest influences from the scale of the study and the heterogeneity of the populations. Given the limited research conducted on Asian patients, further high-quality studies with greater precision are required to validate the predictive capability of the IMPEDE score in this population.

The cumulative incidence of arterial thrombosis, including angina, myocardial infarction, and stroke, within one year of MM diagnosis in the Swedish population was reported to be 3.8% [3]. In a study conducted at Taipei Veterans Hospital, the cumulative incidence of stroke within five years of MM diagnosis was 7.5% [28]. However, in our study, the cumulative incidence of IS within six months of MM diagnosis was 6.3%, which was higher than that reported in previous studies. Although a direct comparison of incidence rates is challenging owing to the variations in the study design contingent upon study objectives, the consistency in the findings that the incidence of IS is higher than that of VTE is noteworthy. Kristinsson et al. (2010, 2012) reported that, compared to controls, patients with MM have a 1.9 times higher incidence of arterial thrombosis, and the occurrence of stroke increases the mortality rate of patients with MM by 4.1 times [3,4]. Despite the high incidence of IS and its significant association with mortality in patients with MM, the tools for identifying high-risk IS patients remain scarce. The existing IS risk prediction tools are primarily designed for use in emergency situations when stroke symptoms manifest or are intended for the general population of patients lacking specialization for patients with MM [8,29]. Furthermore, although one study explored the significant factors associated with stroke occurrence in patients with MM, few studies have quantified the risk using these factors. Considering that IS, like VTE, is a type of thrombosis and does not divide the risk factors for the two diseases, we investigated the potential of using a VTE risk tool in patients with MM to identify high-risk IS groups [2]. Our findings revealed that the incidence of IS in the high-risk VTE group was approximately 22%, which was significantly higher than the approximately 7% incidence in the low-risk group, thus confirming the potential utility of the VTE risk tool in screening high-risk IS groups. Moreover, the high prevalence of VTE history in individuals with IS and the highest VTE history score in the IMPEDE serve as evidence supporting the usefulness of the VTE risk score in identifying the IS risk group. In our study, over half of the patients (1188 of 2358) who developed IS had a history of VTE, demonstrating that the VTE risk score offers sufficient sensitivity for identifying high-risk IS groups. In addition, the C-index exceeded 0.5, confirming the validity of the model.

In our results, the high IMPEDE VTE score group had a risk of IS approximately 3.2 times higher than that in the low-risk group (HR 3.2; 95% CI, 2.8–3.7), which increased to 3.5 times higher when adjusted for sex and age. The occurrence of IS exhibited a significant relationship with sex, age, and the number of comorbidities, with men experiencing an IS risk 1.3 times higher than women (*p* < 0.001), patients > 65 years of age facing a 2.4 times higher risk (*p* < 0.001) than younger patients, and those with a high CCI (≥3) having a 2.7 times higher incidence (*p* < 0.001).

It is notable that despite the valuable findings of our study, there are several limitations in our study. First, BMI, a factor in determining the VTE score, could not be confirmed with the claims data. Consequently, we used an obesity diagnosis code as an alternative indicator of a BMI > 25. A Korean study conducted at a tertiary general hospital found that approximately 27% of patients had a BMI > 25; our study revealed an even higher proportion of patients with an obesity diagnosis code (35%). Utilizing the obesity diagnosis code instead of BMI > 25 might raise concerns regarding the potential overestimation of the VTE risk score. Second, to determine the dosage of dexamethasone for calculating the IMPEDE VTE score, our study included prednisolone, unlike most previous studies that focused solely on dexamethasone. The preliminary data analysis indicated that prednisolone is commonly administered to patients with MM in Korea instead of dexamethasone. While this inclusion may lead to an overestimation of the VTE score, it offers a more precise risk estimate, considering that the increased VTE risk in patients with MM is associated not only with the inherent properties of dexamethasone but also with the high dosage of steroids. Third, the incidence may have been overestimated by relying solely on the diagnostic codes to define the occurrence of VTE or IS. Given the retrospective nature of our study based on the claims data, the clinical context is unknown, potentially leading to an overestimation of the actual occurrences. However, the impact of this limitation may be minimal owing to the study design, which included the implementation of a washout period for the events and defining the occurrence of the first VTE or IS as the endpoint.

To the best of our knowledge, this study is the first attempt to quantify the risk of VTE and IS among patients with MM using a large representative database of the Korean population. Our findings highlight a substantial risk of VTE and IS among MM patients, emphasizing the criticality of early screening and management interventions. Additionally, our investigation into the broader applicability of the IMPEDE VTE score reveals its potential utility in not only stratifying patients based on VTE occurrence but also in assessing the risk of IS occurrence. Consequently, our study provides fundamental data for tailoring treatment strategies aimed at preventing the occurrence of VTE or IS in high-risk groups of patients with MM.

## Figures and Tables

**Table 1:** Baseline characteristics of study population.

Characteristic	Total	Event-Free	VTE	IS	*p*-Value
		*n* (%)	*n* (%)	*n* (%)	
No. of patients	37,168	32,900 (88.5)	1910 (5.1)	2358 (6.3)	
Sex					
Male	20,072 (54.0)	17,648 (53.6)	997 (52.2)	1427 (60.5)	<0.0001
Female	17,096 (46.0)	15,252 (46.4)	913 (47.8)	931 (39.5)	
Age at diagnosis, mean (SD)	65.9 (13.1)	65.4 (13.2)	68.0 (11.7)	71.5 (11.1)	<0.0001
<20	52 (0.1)	51 (0.1)	1 (0.1)	0 (0.0)	<0.0001
20–29	394 (1.1)	381 (1.1)	7 (0.4)	6 (0.3)	
30–39	949 (2.6)	900 (2.6)	26 (1.4)	23 (1.0)	
40–49	2725 (7.3)	2544 (7.3)	100 (5.2)	81 (3.4)	
50–59	6650 (17.9)	6162 (17.9)	275 (14.4)	213 (9.0)	
60–69	10,057 (27.1)	8976 (27.1)	561 (29.4)	520 (22.1)	
70–79	11,053 (29.7)	9469 (29.7)	633 (33.1)	951 (23.9)	
≥80	5288 (14.2)	4417 (14.2)	307 (16.1)	564 (77.4)	
Age ≥ 65, *n* (%)	21,638 (58.2)	18,592 (56.5)	1120 (63.9)	1826 (77.4)	
CCI, mean (SD)	4.2 (3.1)	4.1 (3.1)	4.8 (3.4)	5.5 (3.1)	<0.0001
0	2734 (7.4)	2598 (7.9)	110 (5.8)	26 (1.1)	<0.0001
1	4656 (12.5)	4354 (13.2)	194 (10.2)	108 (4.6)	
2	5454 (14.7)	4967 (15.1)	262 (13.7)	225 (9.5)	
≥3	24,324 (65.4)	20,981 (63.8)	1344 (70.4)	1999 (84.8)	
Comorbidities					
Cerebrovascular disease	6991 (18.8)	4679 (14.2)	385 (20.2)	1927 (81.7)	<0.0001
Myocardial infarction	944 (2.5)	777 (2.4)	55 (2.9)	112 (4.7)	<0.0001
Congestive heart failure	5586 (15.0)	4518 (13.7)	432 (22.6)	636 (27.0)	<0.0001
Peripheral vascular disease	2485 (6.7)	2091 (6.4)	170 (8.9)	224 (9.5)	<0.0001
Paralysis	969 (2.6)	525 (1.6)	74 (2.9)	370 (15.7)	<0.0001
Diabetes Mellitus	17,228 (46.4)	14,715 (44.7)	960 (50.3)	1553 (65.9)	<0.0001
Renal disease	7363 (19.8)	6308 (19.2)	388 (20.3)	667 (28.3)	<0.0001
Liver disease	563 (1.5)	495 (1.5)	23 (1.2)	45 (1.9)	0.1567
Cardiac arrhythmias	3742 (10.1)	2908 (8.8)	293 (15.3)	541 (22.9)	<0.0001
Hypertension	23,221 (62.5)	19,902 (60.5)	1310 (68.6)	2009 (85.2)	<0.0001
Coagulopathy	1853 (5.0)	1522 (4.6)	165 (8.6)	166 (7.0)	<0.0001
Anemia	2725 (7.3)	2383 (7.2)	145 (7.6)	197 (8.4)	0.1224
IMPEDE VTE					
Score, mean (SD)	1.8 (2.8)	1.7 (2.7)	1.8 (3.3)	2.7 (3.8)	<0.0001
(95% CI)	(1.75–1.81)	(1.67–1.74)	(1.66–1.96)	(2.54–2.85)	
IMPEDE score ≥ 8	1084 (2.9)	815 (2.5)	76 (4.0)	193 (8.2)	<0.0001
Body mass index ≥ 25 kg/m^2^	35 (0.1)	31 (0.1)	2 (0.1)	2 (0.1)	<0.0001
Pathologic fracture pelvis/hip/femur	210 (0.6)	184 (0.6)	16 (0.8)	10 (0.4)	<0.0001
VTE history	2438 (6.6)	1022 (3.1)	228 (11.9)	1188 (50.4)	<0.0001
Tunneled line/CVC	3150 (8.5)	2767 (8.4)	216 (11.8)	167 (7.1)	<0.0001
Immunomodulatory drug	5768 (15.5)	5108 (15.5)	486 (25.4)	174 (7.4)	<0.0001
Erythropoiesis-stimulating agent	3412 (9.2)	2919 (8.9)	265 (13.9)	228 (9.7)	<0.0001
Dexamethasone (high dose)	7853 (21.1)	6967 (21.2)	511 (26.8)	375 (15.9)	<0.0001
Dexamethasone (low dose)	13,445 (36.2)	11,919 (36.2)	795 (41.6)	731 (31.0)	<0.0001
Doxorubicin	779 (2.1)	717 (2.2)	47 (2.5)	15 (0.6)	<0.0001
Existing therapeutic warfarin or LMWH use	1637 (4.4)	1023 (3.1)	411 (21.5)	203 (8.6)	<0.0001
Existing prophylactic aspirin or LMWH use	11,973 (32.2)	9933 (30.2)	970 (50.8)	1070 (45.4)	<0.0001

**Table 2:** Multivariable Cox hazards analysis for VTE event.

Variables	Model 1		Model 2		Model 3		Model 4	
	HR (95%CI)	*p*-Value	HR (95%CI)	*p*-Value	HR (95%CI)	*p*-Value	HR (95%CI)	*p*-Value
VTE IMPEDE score ≥ 8	1.54 (1.23–1.94)	0.0002	1.54 (1.23–1.94)	0.0002	1.62 (1.29–2.04)	<0.0001	1.59 (1.26–2.00)	<0.0001
Male			0.95 (0.87–1.04)	0.2816	0.96 (0.87–1.05)	0.3223	0.95 (0.86–1.03)	0.2219
Age ≥ 65					1.40 (1.27–1.53)	<0.0001	1.34 (1.22–1.47)	<0.0001
CCI ≥ 3							1.29 (1.17–1.42)	<0.0001

**Table 3:** Multivariable Cox hazards analysis for IS event.

Variables	Model 1		Model 2		Model 3		Model 4	
	HR (95%CI)	*p*-Value	HR (95%CI)	*p*-Value	HR (95%CI)	*p*-Value	HR (95%CI)	*p*-Value
VTE IMPEDE score ≥ 8	3.23 (2.79–3.75)	<0.0001	3.22 (2.78–3.73)	<0.0001	3.72 (3.21–4.31)	<0.0001	3.47 (2.99–4.02)	
Male			1.33 (1.22–1.44)	<0.0001	1.35 (1.24–1.46)	<0.0001	1.30 (1.20–1.41)	<0.0001
Age ≥ 65					2.80 (2.51–3.05)	<0.0001	2.42 (2.42–2.20)	<0.0001
CCI ≥ 3							2.65 (2.37–2.97)	<0.0001

## Data Availability

The data used in this study are not publicly accessible, primarily because of privacy and ethical constraints. Access to the dataset utilized in this study was restricted to authorized researchers through the Korean National Health Insurance internal networking system.

## Supplementary Materials

The following supporting information can be downloaded from https://www.mdpi.com/article/10.3390/jcm13102829/s1, Table S1. Definitions and codes for variables of the IMPEDE VTE scores.
