# Supplementary material for: The Risk of Venous Thromboembolism and Ischemic Stroke Stratified by VTE Risk Following Multiple Myeloma: A Korean Population-Based Cohort Study
Source: J Clin Med. 2024 May 11;13(10):2829. doi: 10.3390/jcm13102829 (PMC11121838; doi:10.3390/jcm13102829)
Supplement: Supplementary file 1 [file jcm-13-02829-s001.zip › jcm-2987298-supplementary.pdf]

Supplementary

Table S1. Definitions and codes for variables of the IMPEDE VTE scores [1-6].

Variables	Scores	Definition†	Codes
Immunomodulatory drug	+4	ATC codes (-30, 30)	Thalidomide: L04AX02; Lenalidomide: L04AX04; Pomalidomide: L04AX06.
Body mass index ≥25 kg/m2	+1	ICD-10 codes (-365, -1)	Obesity: E66
Pathologic fracture pelvis/hip/femur	+4	ICD-10 codes (-30, -1)	Pelvis: S320, S321, S328; Hip/femur: S720, S721, S724.
Erythropoiesis-stimulating agent	+1	ATC codes (-30, 30)	Methoxy polyethylene glycol-epoetin β: B03XA03; Darbepoetin α: B03XA02.
Dexamethasone (high dose: ≥ 160mg/4weeks)	+4	ATC codes (-30, 30)	D07XB05, H02AB06
Dexamethasone (low dose: < 160mg/4weeks)	+2	ATC codes (-30, 30)	D07XB05, H02AB06
Doxorubicin	+3	ATC codes (-30, 30)	L01DB01
Ethnicity/race: Asian race	-3	All patients	NA
VTE history	+5	ICD-10 codes (-365, -183)	I26, I80, I81, I82, O082, O223, O871, O882
Tunneled line/CVC	+2	Procedure codes (-365, -1)	O1654,O1655,O1656
Existing therapeutic warfarin or LMWH use	-5	ATC codes with ICD-10 codes for AF or VTE (-30, 30)	Warfarin: B01AA03; Enoxaparin: B01AB05; Nadroparin: B01AB06; Dalteparin: B01AB04; Bemiparin: B01AB12; AF: I48; VTE: I26, I80, I81, I82, O082, O223, O871, O882.
Existing prophylactic aspirin or LMWH use	-3	ATC codes without ICD-10 for VTE (-30, 30)	Aspirin: A01AD05, B01AC06, N02BA01; VTE: I26, I80, I81, I82, O082, O223, O871, O882.

Abbreviations: AF, atrial fibrillation; ATC, Anatomical Therapeutic Chemical; ICD-10, International Classification of Diseases 10th; VTE, venous thromboembolism.

† The index date (Day 0) was defined as the first calendar date of chemotherapy.

Reference

1. Chalayer, E., et al., *Prediction of venous thromboembolism in patients with multiple myeloma treated with lenalidomide, bortezomib, dexamethasone, and transplantation: Lessons from the substudy of IFM/DFCI 2009 cohort.* Journal of Thrombosis and Haemostasis, 2022. **20**(8): p. 1859-1867.
2. Covut, F., et al., *Validation of the IMPEDE VTE score for prediction of venous thromboembolism in multiple myeloma: a retrospective cohort study.* British Journal of Haematology, 2021. **193**(6): p. 1213-1219.
3. Jang, D.S., et al., *Stratification of Venous Thromboembolism Risk in Multiple Myeloma and Analysis of the Use of Antithrombotic Agents.* Journal of Korean Society of Health-System Pharmacists, 2023. **40**(2): p. 158-170.
4. Li, A., et al., *Derivation and Validation of a Risk Assessment Model for Immunomodulatory Drug-Associated Thrombosis Among Patients With Multiple Myeloma.* J Natl Compr Canc Netw, 2019. **17**(7): p. 840-847.
5. Li, X., et al., *Development and validation of a new risk assessment model for immunomodulatory drug-associated venous thrombosis among Chinese patients with multiple myeloma.* Thromb J, 2023. **21**(1): p. 105.
6. Sanfilippo, K.M., et al., *Predicting venous thromboembolism in multiple myeloma: development and validation of the IMPEDE VTE score.* American journal of hematology, 2019. **94**(11): p. 1176-1184.
